# Metabolic Profiles of Finishing and Nonfinishing Horses in Uruguayan Raid Competitions

**DOI:** 10.1155/vmi/4217400

**Published:** 2025-11-29

**Authors:** Gimena Brito, Juan Pablo Damián, Pablo Trigo, Gretel Ruprechter

**Affiliations:** ^1^Departamento de Clínicas y Hospital Veterinario, Facultad de Veterinaria, Universidad de la República, Montevideo, Uruguay; ^2^Departamento de Biociencias Veterinarias, Facultad de Veterinaria, Universidad de la República, Montevideo, Uruguay; ^3^Núcleo de Bienestar Animal, Facultad de Veterinaria, Universidad de la República, Montevideo, Uruguay; ^4^IGEVET CONICET CC La Plata, Facultad de Ciencias Veterinarias, Universidad Nacional de la Plata, La Plata, Argentina

**Keywords:** biochemistry, endurance, equine, exercise physiology, long exercise, performance

## Abstract

The Raid Hípico Uruguayo (RHU) is the oldest equestrian endurance sport in Uruguay. A high percentage of horses fail to complete RHU rides. Therefore, the aim of this study was to investigate whether weather conditions (comfort index [CI]), horse experience, and ride distance affect horse performance (finishing ride [FR] or nonfinishing ride [NFR]) in the RHU. An additional objective was to determine whether finishing the ride affects the hematological and biochemical parameters of the horses. This study involved 17 RHU rides over distances of 60–90 km and 284 horses. Blood samples were taken before and after the competition or upon withdrawal (retired or eliminated). The nonfinishing group consisted of 169 horses. Horse performance was associated with CI (*P* < 0.05), but not with ride distance (*P* = 0.33). Horse experience tended to be associated with finishing the ride (*P* = 0.09). Hematocrit, creatine kinase activity, urea, and uric acid concentrations were greater in FR than in NFR horses (*P* < 0.05) and all parameters were affected by the time of sampling (*P* < 0.001), being higher in the postride sample. Weather conditions significantly affected horse performance during RHU competitions, whereas ride distance showed no effect. We observed changes in hematological and biochemical parameters regardless of the horse's performance. Most changes seemed to be caused by a decrease in blood volume, an increase in energy expenditure, and muscle damage that were not the result of metabolic disease but were related to a physiological response to the intensity and duration of exercise.

## 1. Introduction

The Raid Hípico Uruguayo (RHU) is the oldest equestrian endurance sport in Uruguay and is regulated by the Uruguayan Equestrian Federation (FEU). The ride distances vary between 60 and 115 km in only two phases, with the initial phase encompassing two-thirds of the total distance [1]. Winning horses achieve average speeds between 25 and 37 km/h, with peak speeds above 40 km/h. These competitions strictly adhere to minimum speed control, ensuring consistently high minimum speeds throughout the entire ride. Nonwinning horses are allotted 30–60 min (depending on the ride distance) to cross the finish line after the leading horse. These competitions predominantly unfold on hard terrain with firm surfaces, and riders have to maintain a minimum weight of 85 kg. No prior experience is required for horses or riders to participate in any ride.

Marichal et al. [2] found that 61% of RHU participants did not complete the ride, a higher percentage than has been reported in Fédération Equestre Internationale (FEI) endurance rides [3–5]. The primary reason for elimination in the RHU was metabolic disorders (70%), in contrast with FEI endurance rides, where the highest elimination rates were attributed to lameness [3, 4, 6, 7]. Competitions in hot and humid conditions have been related to fluid and electrolyte losses and the inability to regulate temperature and fatigue, leading to the elimination of horses for metabolic reasons [8–10]. The ride distance or previous experience might impact horse performance in endurance rides. Nagy et al. [3] found that more horses completed 80–100 km rides than 120–160 km rides, and long-distance ride experience reduced the risk of elimination for lameness. Understanding why horses are eliminated in RHU endurance competitions and assessing the effects of distance, experience, and weather conditions on performance may improve horse health and welfare.

The high elimination rate in RHU rides is a frequent topic of discussion in public and professional circles. Despite a growing focus on veterinary issues in RHU horses, there is a lack of evidence-based information on metabolic stress during competitions. While knowledge from FEI endurance rides may apply, RHU rides differ in speed and phase length, posing challenges for veterinarians treating metabolic disorders. Prolonged submaximal aerobic exercise, such as RHU rides, requires substantial energy production over a long time [11] and the cardiorespiratory, endocrine, and neuromuscular systems have to work at an elevated level for an unnatural length of time [9, 12]. During these rides, horses are exposed to thermolysis, loss of electrolytes, and catabolism products as a consequence of hemolysis, partial rhabdomyolysis, and energy metabolism [13].

Hematological and biochemical indicators in blood provide insight into how individual horses respond to physical activity, injury, or disease [14]. Identifying metabolic profiles associated with exercise stress may enhance our understanding of the high energy demand in rides like the RHU. Therefore, the objectives of this study were (1) to study whether weather conditions, horse experience, and ride distance affect outcome (finishing or not finishing the ride) and (2) to determine whether finishing the ride affects the hematological and biochemical parameters of the horses.

## 2. Materials and Methods

### 2.1. Rides and Horses

In accordance with the FEU regulations, to be able to participate in these competitions, all horses must be affiliated to the FEU, be at least 6 years old, be up to date with influenza and rhinopneumonitis vaccinations, and show a negative test for equine infectious anemia. Horses may run for the first time at any distance and prior classification is not required. Relevant information about the horses that participated in this study (sex, age, and breed) and their experience (RHU racing experience) was obtained from their passport. Each horse was assigned a unique identification number guaranteeing confidentiality regarding the names of the horses and owners.

The day before the ride, each horse underwent a general examination including locomotor control to detect evidence of claudication. Heart and respiratory rate, rectal temperature, turgor of skin, color and moisture of the mucous membranes, capillary refill time, and bowel sounds were recorded.

In accordance with FEU regulations, the ride consists of two phases in which horses can either be voluntarily retired by the owner or eliminated due to metabolic issues or lameness. After the first phase (that represents two-thirds of the total distance), horses undergo a veterinary check 20 min after arrival; if veterinarians consider the horse unfit to continue the ride due to their metabolic status or lameness, it is eliminated. Elimination for metabolic reasons considers key parameters such as heart rate, color and moisture of mucous membranes, capillary refill time, turgor of skin, and the presence and intensity of gut sounds. Another metabolic reason is the sole presence of a synchronous diaphragmatic flutter. Horses deemed fit to continue then proceed to the gait evaluation. At this time, the horse is eliminated for lameness if it shows consistent gait irregularity at trot. Besides, the judges may disqualify a horse at any time during the ride if it is not fit to continue or if it presents behavior contrary to the ethics of equestrian sport. Once horses have passed the veterinary check, there is a 40-minute compulsory rest period before starting the last phase.

This study included 17 RHU rides of 60, 80, and 90 km (4, 4, and 9 rides, respectively) between March and November 2019. In these 17 RHU rides, a total number of 484 horses started, 30 were retired in the first phase, 154 were eliminated in the veterinary check, 5 horses were retired by the owner having passed the veterinary check, 102 were retired in the second phase, and 193 finished the ride. The ride characteristics by distance are summarized in Table 1. Out of the total number of horses, 284 were included in the present study.

The ride data were entered into a computerized database, where each horse and ride were assigned a unique identification number. The database included information on ambient temperature, humidity, ride distance (km), the number of horses that started, the number of horses that finished, and the performance of each horse. Based on their performance, horses were classified into two groups: those that completed the ride (FR) and those that did not (NFR). The latter group included horses that were either retired or eliminated due to metabolic reasons (EM) or lameness (EL). Additionally, the average speed of the ride (km/h) was recorded.

For each ride, data on ambient temperature (*T*) and relative humidity (HR) were collected from the National Meteorology Service website. The comfort index (CI) was calculated by adding the temperature in degrees Fahrenheit to the relative humidity, expressed as a percentage, following the method outlined by Jones [15]. Subsequently, the calculated CI was categorized into three groups according to Jones [15]: low (CI < 130, where thermoregulation should not be a concern), medium (CI 130–150, where horses will sweat, but should be able to exercise without major problems if normal fluid replacement is allowed), and high (CI > 150, where heat dissipation could be a problem).

### 2.2. Sample Collection, Management, and Laboratory Analysis

Health monitoring is a mandatory practice for horses in RHU rides. Consequently, pre- and postride blood sampling is a routine procedure for horses participating in this type of competition [16]. Blood samples were taken by official veterinarians in the presence of the owners. Blood samples (10 mL) were collected from the jugular vein at two different times during the study. The first set of samples was obtained at rest (preride) on the day before the competition, specifically between 11:00 a.m. and 01:00 p.m., coinciding with the time of veterinary admission, which was 20 h before the start of the ride.

The second set of samples was collected on the day of the ride (postride) from horses that finished the ride before any treatment was administered, as well as from horses that did not finish the ride. Samples were taken within 5 min of elimination/withdrawal from or completion of the ride.

Immediately after extraction, the blood was transferred to tubes with and without EDTA. From the EDTA tube, a microhematocrit tube was filled and then centrifuged at 11,000 rpm for 5 min to obtain the hematocrit (HTO) percentage. The tubes without EDTA were centrifuged at 5000 rpm for 5 min at 20°C, and the resulting serum was transferred to labeled Eppendorf tubes. These samples were then stored under refrigeration (4°C–8°C) for approximately 24 h during the competition and transport. Subsequently, the samples were frozen at −20°C until further analysis at the Laboratorio de Endocrinología y Metabolismo Animal, Facultad de Veterinaria (Universidad de la República). The biochemical parameters measured were total proteins (PT, mg/dL), albumin (ALB, mg/dL), urea (UREA, mg/dL), creatinine (CREA, mg/dL), creatine kinase (CK, U/L), aspartate aminotransferase (AST, U/L), uric acid (UA, mg/dL) and non-esterified fatty acids (NEFAs, mmol/L), which were analyzed on an automatic spectrophotometer (BA200 Biosystems, Spain) using commercial kits and controls (Biosystems, Spain). For all parameters, the coefficient of variation of controls was lower than 10%.

### 2.3. Statistical Analysis

The frequency of animals categorized within each variable (distance, experienced, and CI) was performed by the proc freq of the SAS OnDemand for Academics software (v. 3.1.0, SAS Institute Inc., Cary, NC, USA), and the statistical differences between horses that completed or did not complete the ride were evaluated using a chi-squared test.

The hematological and biochemical parameters were analyzed with proc glimmix of SAS OnDemand for Academics (v. 3.1.0, SAS Institute Inc., Cary, NC, USA), considering as fixed effects the outcome of the ride (FR or NFR), time of sample (pre- vs. postride), and the interaction between both, and as a random effect the animal within the ride, distance, and experience. The CI was included as a covariate. The distribution of the parameters and errors were checked. The model included HTO with a gamma distribution and the other parameters (PT, ALB, AST, CK, CREA, UREA, UA, and NEFA) with a lognormal distribution. Data are presented as mean ± S.E.M. and the level of significance was set at *p* < 0.05.

### 2.4. Ethics Approval

The study protocol was approved by the Comisión de Ética en el Uso de Animales (CEUA) of the Facultad de Veterinaria, Universidad de la República (Uruguay) (approval number 111400-000124-12; 6 March 2013-renewed protocol number 2344/2025). The inclusion of the participants in our study was voluntary and the study information was previously sent to the FEU via email, who approved and communicated the information to their competitors before each ride.

## 3. Results

### 3.1. Descriptive Data

Among the horses that participated in this study (*n* = 284), 216 were crossbreeds, 39 thoroughbreds, 14 Anglo-Arabians, 14 Arabian horses, and 1 had no information. Other recorded animal characteristics such as sex, experience, and age are summarized in Table 2.

Considering the climate index, *n* = 57 horses participated in rides with a low CI, *n* = 153 with a medium CI, and *n* = 74 with a high CI. The average speed of the winning horses was 33.77 ± 2.96, 27.65 ± 0.28, and 27.57 ± 1.05 km/h for 60, 80, and 90 km, respectively. From the total number of horses sampled, *n* = 115 (40.5%) finished the ride and *n* = 169 (59.5%) did not finish the ride. The frequency of horses retired/eliminated for metabolic reasons was greater than those retired/eliminated for lameness (eliminated for metabolic reason = 68.6% (*n* = 116) (first phase *n* = 8, veterinary control *n* = 61, and second phase *n* = 47) versus eliminated for lameness = 31.4% (*n* = 53) (first phase *n* = 4, veterinary control *n* = 45, and second phase *n* = 4); *p* < 0.001).

### 3.2. Impact of Climatic Conditions, Ride Distance, and Experience on the Performance of the Horses

The descriptive data of horses that FR or NFR according to ride distance, experience, and CI are shown in Table 3. The frequency of animals FR or NFR was associated with the CI (*p* < 0.01), but not with the ride distance (*p* = 0.33, Table 3). In rides with a low CI, a higher percentage of animals finished the ride, while in rides with a high CI, a higher percentage of animals did not finish the ride (Table 3). Experience tended to be associated with finishing or not (*p* = 0.09): more experienced than unexperienced horses finished the rides.

### 3.3. Hematological and Biochemical Parameters of Horses That Finished and Did Not Finish the Ride

As shown in Table 4, there were no significant differences in any of the preride samples between NFR and FR horses. In contrast, all parameters were significantly affected by sampling time (*p* < 0.001), with values consistently higher in the postride samples. There was an interaction between performance (FR or NFR) and the time of sampling for HTO, ALB, CK, CREA, UREA, and UA (*p* < 0.05) (Table 4). In the postride samples, the HTO, CK, UREA, and UA concentrations were greater in FR horses than in NFR horses, but no differences were found in ALB and CREA concentrations (Table 4).

## 4. Discussion

The RHU is an equestrian endurance sport in which riders measure their resistance and that of their horses, covering long distances in only two phases. Many of the participants compete to improve their previous performance, with the only objective of crossing the finish line; however, our data show that 59.5% of the riders did not finish the ride. These data agree with Marichal et al. [2] and exceed those reported for FEI endurance by Nagy et al. (51.4%) [3], Muñoz et al. (38.6%) [4], and Legg et al. (39%) [5]. One possible reason for this could be the higher speeds, greater weight of rider/riding equipment, and fewer stages of RHU competitions. Additionally, there were differences in the reason for elimination. In FEI endurance, lameness was the most common cause of elimination, followed by elimination for metabolic reasons (69.2% and 23.5%, respectively [17]). In our study, 31.4% of horses were eliminated due to lameness and 68.6% due to metabolic reasons, highlighting the need for a correct metabolic management of RHU horses. Much of the difference between these disciplines may be due to differences in competition formats. RHU rides are shorter in both distance and duration compared to endurance rides. Additionally, the speed is higher for all participants, particularly for the nonleading horses that complete the ride. The higher intensity of RHU rides, combined with their shorter duration, may account for the results observed in this study.

One of our aims was to determine whether weather conditions, distance, and experience of horses affected the performance of horses in RHU competitions. As expected, the CI affected horse performance in RHU rides. According to Whiting [18], horses running in conditions of excessive heat and humidity tend to exhibit clinical signs of exhaustion earlier than in more temperate climates, leading to a higher rate of eliminations. CI seems to be a sensitive indicator of this phenomenon. Some authors have also described this in endurance and eventing horses [9, 10, 19]. However, in RHU competitions, Marichal et al. [2] did not find an association between CI and the horse finishing or not finishing the ride.

On the other hand, the distance did not affect horse performance in RHU rides, in accordance with Nagy et al. [3], who concluded that in endurance rides, the distance of the ride was not the determining factor for the occurrence of exhaustion. Our results suggest that the determining factor for finishing RHU rides was not directly associated with the ride distance but could be linked to the intensity of training and the fitness of the horses. Furthermore, in our study, more experienced horses tended to complete the ride, which agrees with previous findings showing that horses that finished had more years of experience in endurance competition compared to those that were eliminated [3, 20]. The sum of these factors suggests that riders and owners may help prevent health issues in their horses not only through proper training and nutrition but also by paying special attention to the condition of their horse during the ride when the animal lacks experience at a particular distance. However, future studies with a larger sample size should be designed to conclusively investigate the effect of the horses' experience on metabolic responses.

Hematological and biochemical variables are known to provide information on how horses respond to physical activity, injury, or disease [14]. Identifying the metabolic profiles associated with exercise may improve our understanding of the high energy demand and provide a picture of the changes that occur in horses performing at events like the RHU. According to our knowledge, this is the first study evaluating the hematological and biochemical parameters in RHU horses and comparing horses that finished the ride or did not finish the ride. The preride values of all parameters in both FR and NFR were within the normal range for horses at rest [13, 21–23]. On the other hand, all parameters were affected by the time of sampling, being greater in the postride sample. These results highlight that an RHU ride consistently modifies the metabolic profiles of horses, regardless of the performance in the competition. Likewise, it has been reported that endurance rides consistently modify the plasma metabolic profiles of horses after the ride regardless of individual performance in the competition, concluding that the metabolic profile largely depends on the effect of prolonged exercise [24].

Many studies performed in FEI endurance rides [9, 12, 21–23, 25–27] found higher biochemical concentrations in several parameters in horses that did not finish the ride. However, in horses competing in the RHU, the effect of distance seemed to be more important for variables such as HTO, CK, UREA, UA, and NEFA, and the effect of time for HTO, CK, UREA, and UA. The rest of the parameters did not vary significantly between both groups of horses. NFR horses experienced lower levels of dehydration and muscle stress compared to FR horses. This may be attributed to the fact that NFR horses covered a shorter distance before being eliminated from the ride. Additionally, horses eliminated due to lameness likely exerted themselves less than those that completed the full ride distance. This highlights the physical demands of endurance racing and how factors like distance covered and reasons for elimination can impact the physiological stress experienced by horses during the event.

As has been previously described for horses competing in endurance rides [21, 22, 25, 28, 29] and the RHU [2], the HTO was higher at the end of the ride. In our study, HTO increased by 27.5% in NFR horses and 38.5% in FR horses. These changes are consistent with hemoconcentration due to splenic contraction and erythrocyte mobilization, fluid redistribution, and fluid losses related to sweating [27, 30, 31]. Generally, an increase in HTO is accompanied by an increase in PT, but not in this case. On the other hand, the concentration of postride ALB increased in both groups, similar to other studies [25, 32]. According to Gondin et al. [33], fluid is displaced from the intravascular space to the interstitial and intracellular space during exercise in horses, which may lead to an increase in ALB serum concentrations according to the length and intensity of the type of exercise.

The decrease in plasma volume described above is proportional to the intensity and duration of the effort and leads to lower renal perfusion during the ride. As a result, UREA and CREA concentrations are increased after RHU rides. The reduction in perfusion and glomerular filtration rate is likely due to the redistribution of blood flow toward the active muscles. This is consistent with studies on horses competing in endurance rides, which concluded that UREA [23] and CREA [12] concentration increased in horses after prolonged effort. The increase in postride CREA concentration was the same in both groups. This agrees with Barton et al. [22], who found no difference between horses that competed over different distances. On the other hand, UREA concentrations in FR horses were higher than those in NFR, associated with greater hemoconcentration due to fluid imbalance in the FR group.

After the ride, the most significant increase in enzymatic activity was observed in CK, an indicator of altered sarcolemma permeability [34]. CK elevations in endurance horses, especially when accompanied by increased HTO and UA levels, may signal exhausted horse syndrome, posing serious risks to the animal [35]. However, most endurance horses experience increased postcompetition CK due to temporary sarcolemma alterations, which are not associated with permanent muscle damage or impaired performance [34, 36, 37]. In our study, CK increased in both FR and NFR horses, although the increase was more pronounced in FR horses. Similarly, muscle damage was not detected during preride veterinary examinations in horses with elevated CK activity before the ride. This agrees with Kerr and Snow [38] who did not find a correlation between CK, AST, and horse performance, noting that elevated CK and AST activities were not clinically important. In contrast, no difference in AST concentration was found between FR and NFR horses, likely because AST plasma activity rises 12–48 h after muscle injury [39–42].

Fatty acid (either released from triglycerides or circulating in the blood as NEFAs) metabolism plays a significant role in the energy supply, enabling increased exercise duration [43]. During prolonged, low-intensity exercise in horses, aerobic metabolism predominantly utilizes NEFA as a primary source for ATP regeneration. In the present study, the increase in NEFA in FR horses suggests a greater mobilization of fatty acids from adipose tissue and increased availability for energy production. Moreover, increased utilization of lipid metabolism in these animals could have contributed to an improved performance by delaying fatigue and producing more metabolic water per generated unit of energy.

In FR horses, the pronounced elevation of NEFAs is evidence of enhanced lipid mobilization to fuel prolonged aerobic energy demands, a critical adaptation for endurance exercise. Concurrently, the marked increase in UA levels reflects significant anaerobic metabolic contributions, likely arising from high-intensity efforts or cumulative muscle stress, which promote nucleotide degradation and oxidative byproducts. This dual activation of aerobic and anaerobic pathways highlights the metabolic versatility that is required to sustain the demands of RHU competitions, where horses must balance sustained endurance with intermittent high-intensity efforts. The elevated NEFA concentrations indicate enhanced fatty acid oxidation, optimizing energy efficiency and delaying glycogen depletion, while the rise in UA signals transient anaerobic activity and oxidative stress, potentially linked to purine catabolism during ATP turnover. Notably, the metabolic response observed in FR horses, characterized by both aerobic and anaerobic components, suggests a physiological capacity to manage energetic trade-offs under strenuous conditions. These findings align with studies in endurance horses, where balanced substrate utilization correlates with performance success [23, 44, 45]. In fact, the RHU's different structure (longer phases and higher speeds) may amplify these metabolic shifts, as rapid energy demands require concurrent aerobic efficiency and anaerobic recruitment.

The present study has certain limitations that need to be emphasized. Enrollment in the study was voluntary and did not include all participating horses. This represents a possible selection bias that is extremely difficult to avoid in cross-sectional field studies. Another limitation was a lack of standardized feeding or detailed nutritional surveys, although most horses were fed balanced diets for endurance horses. Further studies are needed to investigate the variation in parameters and the influence of sampling time and to determine the time for recovery to basal values to define the possible effects on the metabolism of the athletic horse. Of course, it would have been better to sample the horses at the veterinary check and compare groups of horses at the same distance. However, the rules of the competition do not allow the collection of blood samples during the ride. Another limitation is the difference in training status of the horses. Level of training can therefore be a confounder in our study. Some horses may have been in suboptimal physical condition, while others—despite being fit—were overexerted. Their physiological responses would probably be different. However, these elements open up new questions to be answered in future studies.

This study seems to indicate that, based on the parameters we analyzed, RHU horses that finished the ride cope well with the stress and physical demands of the competition. In the group of animals that did not finish the ride, a higher proportion of animals was eliminated for metabolic reasons than has been described for FEI endurance competitions. Therefore, we expected the indicators for dehydration, muscle injury, and fatigue to be higher in this group, but the specific conditions of the RHU competitions, such as speed and distance, are more likely behind this difference.

## 5. Conclusion

Climatic conditions significantly affect the performance of horses during RHU competitions, whereas ride distance does not affect performance. During RHU rides, changes occur in hematological and biochemical parameters—in this study increased HTO, ALB, CK, UREA, CREA, and UA—regardless of the performance of the horse; therefore, changes in these parameters are not caused by metabolic disease but are related to a physiological response to the intensity and duration of exercise.

The findings of this study suggest that Uruguayan raid organizers and veterinarians should consider implementing more exhaustive monitoring protocols during unfavorable weather conditions.

## Figures and Tables

**Table 1:** Number of horses that started and finished the ride or were voluntarily retired or eliminated at Raid Hípico Uruguayo competitions in 2019 in 17 rides.

Ride (km)	Started^∗^ (*n*)	Abandoned/eliminated metabolic reasons (*n*)			Abandoned/eliminated lameness (*n*)			Voluntarily retired (*n*)	Finished (*n*)
		First phase	Vet check	Second phase	First phase	Vet check	Second phase		
60	83	2	14	11	3	17	0	1	35
80	147	6	25	29	2	14	1	3	67
90	254	12	47	54	5	37	7	1	91
Total	484	20	86	94	10	68	8	5	193

^∗^The same horse could be included in different rides.

**Table 2:** Descriptive data on sex, experience, and age of the sampled horses that participated in 17 Raid Hípico Uruguayo competitions in 2019.

Ride (km)	Horses (*n*)^∗^	Sex (*n*)		Racing experience (*n*)		Average age (years)
		Gelding	Female	Yes	No	
60	47	33	14	24	23	8.3
80	76	59	17	52	24	9
90	161	114	47	123	38	8.6
Total	284	206	78	199	85	8.6

^∗^The same horse could be included in different rides.

**Table 3:** Descriptive data of horses that finished (FR) or did not finish the ride (NFR) according to the ride distance (km), the experience of the horses, and the comfort index.

	FR (*n* = 115)	NFR (*n* = 169)	*p* value^a^
Distance (km)			
** **60	23 (20%)	24 (14%)	0.33
** **80	32 (28%)	44 (26%)	
** **90	60 (52%)	101 (60%)	
Experience			
** **No	28 (24%)	57 (34%)	0.09
** **Yes	87 (76%)	112 (66%)	
Comfort index			
** **Low	32 (28%)	25 (15%)	0.01
** **Medium	61 (53%)	92 (54%)	
** **High	22 (19%)	52 (31%)	

^a^Chi-squared test.

**Table 4:** Hematological and biochemical parameters before and after the ride (mean ± S.E.M.) in horses that finished (FR) and did not finish (NFR) the ride.

	NFR		*p* value	FR		*p* value
	Preride	Postride		Preride	Postride	
HTO (%)^∗^	39.7 ± 0.41	50.6 ± 0.41^ǂ^	< 0.001	38.7 ± 0.49	54.4 ± 0.49^ǂ^	< 0.001
PT (mg/dL)	6.31 ± 0.26	7.51 ± 0.26	< 0.001	6.27 ± 0.26	7.65 ± 0.26	< 0.001
ALB (mg/dL)^∗^	3.18 ± 0.03	3.86 ± 0.03	< 0.001	3.15 ± 0.03	3.94 ± 0.03	< 0.001
AST (U/L)	224.3 ± 11.8	289.7 ± 11.7	< 0.001	225.6 ± 14.2	298.9 ± 14.2	< 0.001
CK (U/L)^∗^	161 ± 107	1189 ± 109^ǂ^	< 0.001	138 ± 129	1508 ± 132^ǂ^	< 0.001
CREA (mg/dL)^∗^	1.71 ± 0.15	2.50 ± 0.15	< 0.001	1.69 ± 0.15	2.74 ± 0.15	< 0.001
UREA (mg/dL)^∗^	35.77 ± 1.84	46.05 ± 1.84^ǂ^	< 0.001	35.68 ± 1.85	51.07 ± 1.85^ǂ^	< 0.001
UA (mg/dL)^∗^	10.23 ± 1.55	33.42 ± 1.55^ǂ^	< 0.001	9.64 ± 1.68	45.28 ± 1.69^ǂ^	< 0.001
NEFA (mmol/L)	0.343 ± 0.084	1.347 ± 0.084	< 0.001	0.404 ± 0.087	1.926 ± 0.088	< 0.001

*Note:* Variables marked with an asterisk (^∗^) indicate an interaction (*P* < 0.05) between performance (FR or NFR) and the time of sampling (pre- or postride).

Abbreviations: ALB, albumin; AST, aspartate transaminase; CK, creatine kinase; CREA, creatinine; HTO, hematocrit; NEFAs, non-esterified fatty acids; PT, total serum protein; UA, uric acid.

^ǂ^Significant difference (*p* < 0.05) between FR and NFR in the postride samples.

## Data Availability

The datasets used and/or analyzed during the current study are available from the corresponding author upon request.

## References

[1] Brito G., Damián J. P., Suárez G., Ruprechter G., Trigo P. (2023). Characterization of Raid Hipico Uruguayo Competencies by Ride Type: Causes of Death and Risk Factors. *Animals*.

[2] Marichal G., Trigo P., Soto C., Meikle A., Suárez G. (2023). Hydroelectrolytic and Acid-Base Parameters After 80 to 115 Km Endurance Races (Raid Uruguayo) and their Association with the Comfort Index. *Animals*.

[3] Nagy A., Murray J. K., Dyson S. J. (2014). Horse-rider-venue- and environment-related Risk Factors for Elimination from Federation Equestre Internationale Endurance Rides due to Lameness and Metabolic Reasons. *Equine Veterinary Journal*.

[4] Muñoz-Alonzo L., Silva A. B., Leal J. C., Luengo M. B. (2016). Motivos de Eliminación en Competencias de Enduro Internacional, Categoría Jinete Adulto, en Chile (2007–2014). *Rev Inv Vet Perú*.

[5] Legg K. A., Weston J. F., Gee E. K., Bolwell C. F., Bridges J. P., Rogers C. W. (2019). Characteristics of Endurance Competitions and Risk Factors for Elimination in New Zealand During Six Seasons of Competition (2010/11–2015/16). *Animals*.

[6] Nagy A., Murray J., Dyson S. (2010). Elimination from Elite Endurance Rides in Nine Countries: a Preliminary Study. *Equine Veterinary Journal*.

[7] Bloom F., Draper S., Bennet E., Marlin D., Williams J. (2022). Risk Factors for Lameness Elimination in British Endurance Riding. *Equine Veterinary Journal*.

[8] Marlin D. J., Scott C. M., Schroter R. C (1996). Physiological Responses in Nonheat Acclimated Horses Performing Treadmill Exercise in Cool (20°C/40%RH), Hot Dry (30°C/40%RH) and Hot Humid (30°C/80%RH) Conditions. *Equine Veterinary Journal*.

[9] Flaminio M. J., Rush B. R. (1998). Fluid and Electrolyte Balance in Endurance Horses. *Veterinary Clinics of North America: Equine Practice*.

[10] Poškienė I., Juozaitienė V., Gruodytė R., Antanaitis R. (2020). The Effect of 60 Km Endurance Exercise on Serum Electrolytes and acid–base Balance in the Žemaitukai Horses. *Acta Veterinaria Brno*.

[11] Acosta R. (1995). *Manual Para Veterinarios En Raid*.

[12] Schott I. I. H. C., Marlin D. J., Geor R. G. (2006). Changes in Selected Physiological and Laboratory Measurements in Elite Horses Competing in a 160 Km Endurance Ride. *Equine Veterinary Journal*.

[13] Muñoz A., Riber C., Trigo P., Castejón‐Riber C., Castejón F. M. (2010). Dehydration, Electrolyte Imbalances and renin-angiotensin-aldosterone-vasopressin Axis in Successful and Unsuccessful Endurance Horses. *Equine Veterinary Journal*.

[14] Klobučar K., Vrbanac Z., Gotić J., Bojanić K., Bureš T., Brkljača Bottegaro N. (2019). Changes in Biochemical Parameters in Horses During 40 Km and 80 Km Endurance Races. *Acta Veterinaria*.

[15] Jones S. Horseback Riding in the Dog Days. *Anim Sci e-News2009*.

[16] (2024). FEU Federación Ecuestre Del Uruguay. http://www.federacionecuestreuruguaya.com.uy/documentacion.html.

[17] Nagy A., Dyson S., Murray J. K. (2012). A Veterinary Review of Endurance Riding as an International Competitive Sport. *The Veterinary Journal*.

[18] Whiting J., Robinson N. E., Sprayberry K. A. (2009). The Exhausted Horse. *Current Therapy in Equine Medicine*.

[19] Andrews F. M., Ralston S. L., Williamson L. H., Maykuth P. L., White S. L., Provenza M. (1995). Weight Loss, Water Loss and Cation Balance During the Endurance Test of a 3-day Event. *Equine Veterinary Journal*.

[20] Schott I. I. H. C., McGlade K. S., Hines M. T., Petersen A. (1996). Bodyweight, Fluid and Electrolyte, and Hormonal Changes in Horses that Successfully Completed a 5 Day, 424kilometer Endurance Competition. *Pferdeheilkunde Equine Medicine*.

[21] Schott H. C., McGlade K. S., Molander H. A., Leroux A. J., Hines M. T. (1997). Body Weight. Fluid, Electrolyte, and Hormonal Changes in Horses Competing in 50- and 100-mile Endurance Rides. *American Journal of Veterinary Research*.

[22] Barton M. H., Williamson L., Jacks S., Norton N. (2003). Body Weight, Hematologic Findings and Serum and Plasma Biochemical Findings of Horses Competing in a 48-83-or 159-km Endurance Ride Under Similar Terrain and Weather Conditions. *American Journal of Veterinary Research*.

[23] Castejón F., Trigo P., Muñoz A., Riber C. (2006). Uric Acid Responses to Endurance Racing and Relationships with Performance, Plasma Biochemistry and Metabolic Alterations. *Equine veterinary journal. Supplement*.

[24] Le Moyec L., Robert C., Triba M. N. (2014). Protein Catabolism and High Lipid Metabolism Associated with Long Distance Exercise Are Revealed by Plasma NMR Metabolomics in Endurance Horses. *PLoS One*.

[25] Rose R. J., Hodgson D. R., Sampson D., Chan W. (1983). Changes in Plasma Biochemistry in Horses Competing in a 160 Km Endurance Ride. *Australian Veterinary Journal*.

[26] Sloet van Oldruitenborgh-Oosterbaan M., Wensing T., Barneveld A., Breukink H. J. (1991). Heart Rate, Blood Biochemistry and Performance of Horses Competing in a 100 Km Endurance Ride. *The Veterinary Record*.

[27] Fielding C. L., Magdesian K. G., Rhodes D. M., Meier C. A., Higgins J. C. (2009). Clinical and Biochemical Abnormalities in Endurance Horses Eliminated from Competition for Medical Complications and Requiring Emergency Medical Treatment: 30 Cases (2005–2006). *Journal of Veterinary Emergency and Critical Care*.

[28] Carlson G. P., Mansmann R. A. (1974). Serum Electrolyte and Plasma Protein Alterations in Horses Used in Endurance Rides. *Journal of the American Veterinary Medical Association*.

[29] Rose R. J. (1982). Haematological Changes Associated with Endurance Exercise. *The Veterinary Record*.

[30] Rose R. J., Arnold K. S., Church S., Paris R. (1980). Plasma and Sweat Electrolyte Concentrations in the Horse During Long Distance Exercise. *Equine Veterinary Journal*.

[31] Freeman D. E., Mooney A., Giguère S., Claire J., Evetts C., Diskant P. (2021). Effect of Feed Deprivation on Daily Water Consumption in Healthy Horses. *Equine Veterinary Journal*.

[32] Assunção P., Barbosa T., Yonezawa L. (2019). Acute-Phase Protein Profile in Horses Subjected to Different Exercise Protocols. *Canadian Journal of Veterinary Research*.

[33] Gondin M. R., Foz N. S. B., Pereira M. C. (2013). Acute Phase Responses of Different Positions of High-Goal (Elite) Polo Ponies. *Journal of Equine Veterinary Science*.

[34] Valverg S., Jönsson L., Lindholm A., Holmgren N. (1993). Muscle Histopathology and Plasma Aspartate Aminotransferase, Creatine Kinase and Myoglobin Changes with Exercise in Horses with Recurrent Exertional Rhabdomyolysis. *Equine Veterinary Journal*.

[35] Hyyppä S., Pösö A. R., Hinchliff K. W., Kaneps A. J., Geor R. J. (2004). Metabolic Diseases of Athletic Horses. *Equine Sports Medicine and Surgery: Basic and Clinical Sciences of the Equine Athlete*.

[36] Barrey E., Mucher E., Robert C., Amiot F., Gidrol X. (2010). Gene Expression Profiling in Blood Cells of Endurance Horses Completing Competition or Disqualified due to Metabolic Disorder. *Equine Veterinary Journal*.

[37] Serteyn D., Sandersen C., Lejeune J. (2010). Effect of a 120 Km Endurance Race on Plasma and Muscular Neutrophil Elastase and Myeloperoxidase Concentrations in Horses. *Equine Veterinary Journal*.

[38] Kerr M. G., Snow D. H., Snow D. H., Persson S. G. B., Rose R. J. (1983). Plasma Enzyme Activities in Endurance Horses. *Equine Exercise Physiology*.

[39] Cardinet G. H., Fowler M. E., Tyler W. S. (1963). Heart Rates and Respiratory Rates for the Evaluating Performance in Horses During Endurance Trial Ride Competition. *Journal of the American Veterinary Medical Association*.

[40] Siciliano P. D., Lawrence L. M., Danielsen K., Powell D. M., Thompson K. N. (1995). Effect of Conditioning and Exercise Type on Serum Creatine Kinase and Aspartate Aminotransferase Activity. *Equine Veterinary Journal*.

[41] Muñoz A., Riber C., Santisteban R., Lucas R. G., Castejon F. M. (2002). Effect of Training Duration and Exercise on blood-borne Substrates, Plasma Lactate and Enzyme Concentrations in Andalusian, Anglo-Arabian and Arabian Breeds. *Equine Veterinary Journal*.

[42] Muñoz A., Cuesta I., Riber C., Gata J., Trigo P., Castejón F. M. (2006). Trot Asymmetry in Relation to Physical Performance and Metabolism in Equine Endurance Rides. *Equine veterinary journal. Supplement*.

[43] Snow D. H., Mason D. K., Ricketts S. W., Douglas T. A., Snow D. H., Persson S. G. B., Rose R. J. (1983). Post-Race Blood Biochemistry in Thoroughbreds. *Equine Exercise Physiology*.

[44] Schuback K., Essén-Gustavsson B. (1998). Effects of Training and Time of Day of Blood Sampling on the Variation of Some Common Haematological Parameters in the Normal Throroughbred Racehorses. *Equine Veterinary Journal*.

[45] Adamu L., Noraniza M. A., Rasedee A., Bashir A. (2012). Metabolic Responses in Endurance Horses During Racing in Relation to Uric Acid Profile, Leucocytes, Heart Rate and Plasma Biochemical Parameters. *Vet Med*.

